# Image diagnosis: Eisenmenger’s syndrome in patients with simple congenital heart disease

**DOI:** 10.1186/s12872-020-01489-y

**Published:** 2020-04-23

**Authors:** Haisong Bu, Xueyang Gong, Tianli Zhao

**Affiliations:** The Department of Cardiovascular Surgery, The Second Xiangya Hospital, Central South University, 139 Renmin Central Road, Changsha, Hunan 410011 People’s Republic of China

**Keywords:** Pulmonary arterial hypertension, Congenital heart disease, Eisenmenger’s syndrome, Heart and lung transplantation

## Abstract

**Background:**

Early identification of congenital heart disease (CHD) allows detection of the pulmonary arteriopathy in an early stage, and timely shunt closure can permanently reverse pulmonary arterial hypertension (PAH). However, surgical correction is not recommended in patients with irreversible PAH. Herein we report our experience about Eisenmenger’s syndrome in simple CHD.

**Case presentation:**

From January 2017 to November 2018, a total of 8 CHD patients (3 ventricular septal defects (VSD), 2 atrial septal defects (ASD), and 3 patent ductus arteriosus (PDA), median age, 15.5 years [range, 3–18 years]) with PAH were detected by chest X-ray, electrocardiogram, transthoracic echocardiography (TTE), computed tomographic angiography (CTA) and cardiac catheterization. The median defect diameter, pulmonary artery pressure (PAP), pulmonary vascular resistance (PVR) were 16.5 mm (range, 3–30 mm), 75 mmHg (range, 60–86 mmHg), and 16 Woods units (range, 12–19 Woods units), respectively. Here, we report the representative cases of three types of simple CHD with irreversible PAH. The surgical correction was not performed in all patients who had fixed PAH and were referred to medical treatment.

**Conclusions:**

PAH in CHD can be reversed by early shunt closure, but this potential is lost beyond a certain point of no return. This article highlights the essence of enhancing the level of healthcare and services in Chinese rural areas. Failure to accurately and timely assess PAH will delay effective treatment past optimal treatment time, and even lead to death.

## Background

Pulmonary arterial hypertension (PAH) is a lethal syndrome characterized by increased PAP, PVR and normal pulmonary capillary wedge pressure [1]. Clinical presentation predominantly comprises symptoms of resulting right heart failure [2]. These symptoms however, are preceded by a progressively obstructive arteriopathy that may be clinically silent for many prior years [3]. Echocardiography is the mainstay of non-invasive diagnostic tool during the early screening that depicts pulmonary hypertension or right heart overload. However, the diagnosis of pulmonary hypertension can be confirmed only by right heart catheterization.

Congenital heart disease (CHD) is currently the common type of congenital malformation in infants [4, 5], and PAH presents with unique features in this disease. In these patients, the arteriopathy is triggered by increased pulmonary blood flow resulting from a left-to-right shunt due to intracardiac or extracardiac defect. Early identification of the cardiac defect allows the detection of the pulmonary arteriopathy in an early stage, and can avoid pulmonary vascular disease by repairing CHD [6]. However, the beneficial effects of shunt closure seem lost after a certain point of no return, after which even accelerated PAH progression may occur months to years after surgery [7]. These observations underscore the critical importance of early and accurate detection of this ‘window for reversibility’ in patients with PAH-CHD. Herein we report our experience about irreversible PAH in children with three types of simple CHD.

## Case presentation

We enrolled 8 patients with simple CHD from Second Xiangya Hospital between January 2017 and November 2018, including 2 male and 6 female patients (median age, 15.5 years [range, 3–18 years]; median body weight 39 kg [range, 12–45 kg]). The median defect diameter, pulmonary artery pressure (PAP), pulmonary vascular resistance (PVR) were 16.5 mm (range, 3–30 mm), 75 mmHg (range, 60–86 mmHg), and 16 Woods units (range, 12–19 Woods units), respectively. The detailed information of these patients is shown in Table 1. The CHD was detected by chest X-ray, electrocardiogram, TTE (Vivid™ E9; GE Healthcare, Little Chalfont, United Kingdom), CTA and cardiac catheterization.

**Table 1:** Patient characteristics

Variable	Values
Total number (*n*)	8
Male/female (*n*)	2/6
Median age (years)	15.5 (range, 3.0–18.0)
Median weight (kg)	39.0 (range, 12.0–45.0)
Types of CHD	
ASD (n)	2
VSD (n)	3
PDA (n)	3
Defect diameter (mm))	16.5 (range, 3.0–30.0)
Mean PAP (mmHg)	75.0 (range, 60.0–86.0)
Mean PVR (Wood)	16.0 (range, 12.0–19.0)

Values are presented as median

*ASD*: atrial septal defect, *VSD* ventricular septal defect, *PDA*: patent ductus arteriosus, *PAP* pulmonary artery pressure, *PVR* pulmonary vascular resistance

### ASD associated PAH

**Fig. 1:**
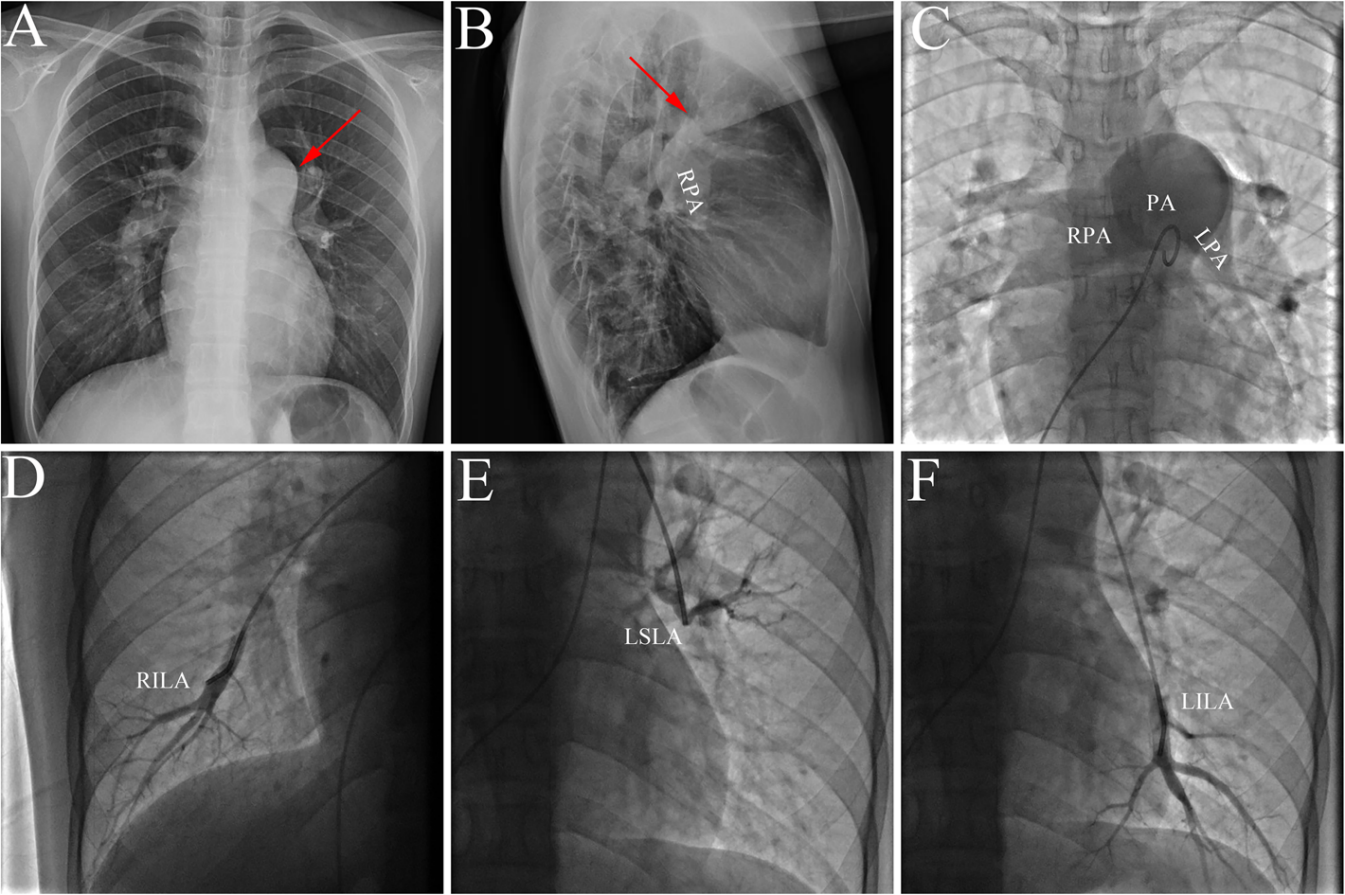
The representative image of PAH-ASD. **a** and **b**: Chest X-ray showed cardiomegaly and a bulging pulmonary artery at the upper left cardiac border; **c**, **d**, **e** and **f**: Pulmonary arteriography showed reduced pulmonary capillary filling. PAH: pulmonary arterial hypertension; ASD: atrial septal defect; PA: pulmonary artery; RPA: right pulmonary artery; LPA: left pulmonary artery; RILA: right inferior lobe artery; LSLA: left superior lobe artery; LILA: left inferior lobe artery

A 17-year-old Chinese girl with a 10-year history of heart murmur and progressive dyspnea was referred to our institution for percutaneous closure of ASD. Physical examination revealed a reduced oxygen saturation in the upper and lower extremity of 90 and 89%, respectively. A loud P2 without a systolic murmur was audible at the second left intercostal space. Electrocardiogram demonstrated right ventricular hypertrophy. Chest X-ray evaluation will show cardiomegaly and a bulging pulmonary artery at the upper left cardiac border (Fig. 1a and b, arrows). TTE revealed a large ASD (25 mm) and PAH (55 mmHg). A right heart catheterization revealed severe fixed PAH with a mean PAP of 75 mmHg and PVR of 12 Woods units. Pulmonary arteriography showed that the degree of pulmonary capillary filling decreased (Fig. 1c, d, e and f). Since the patient had fixed PAH, the surgical correction was not performed.

### VSD associated PAH

**Fig. 2:**
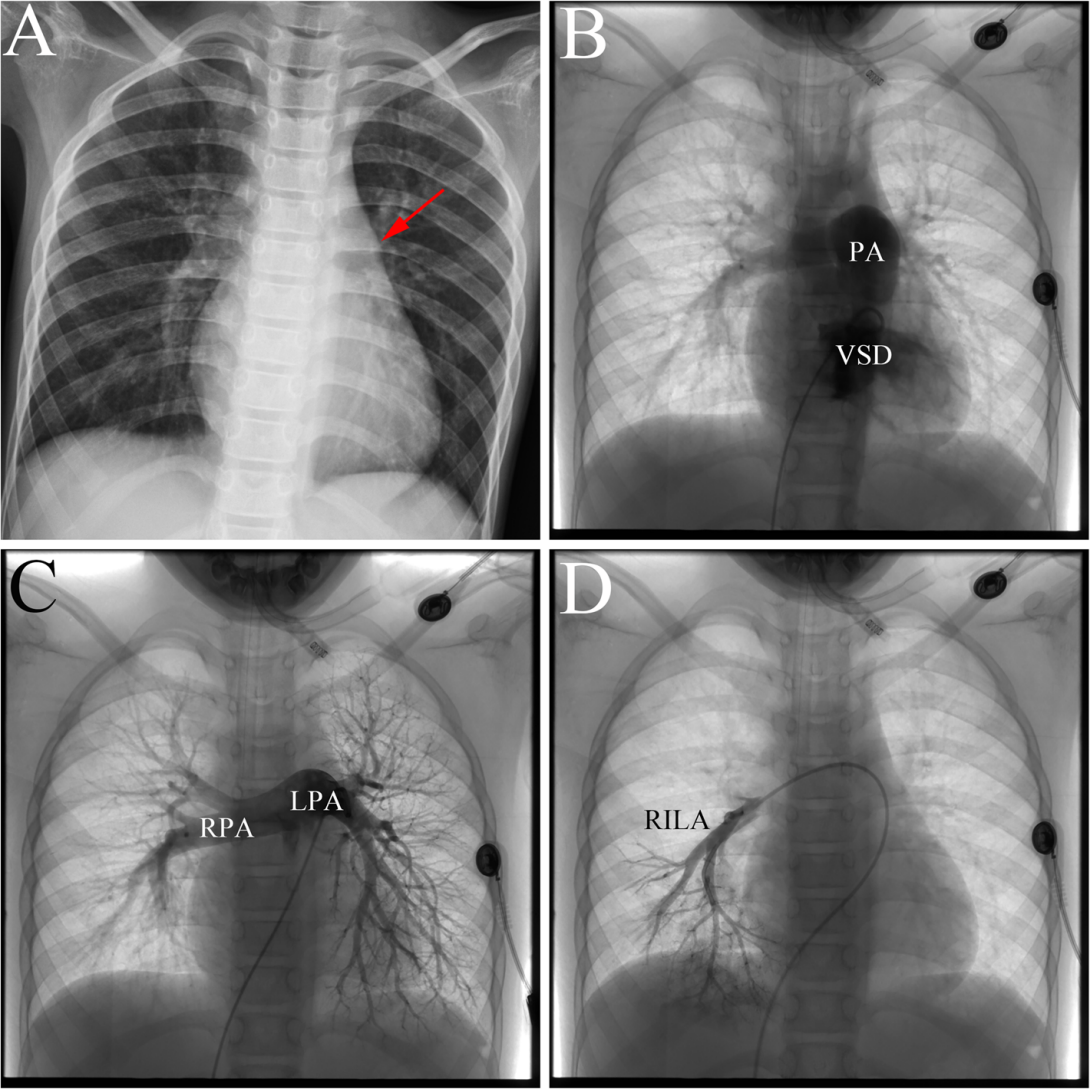
The representative image of PAH-VSD. **a**: Chest X-ray confirmed a bulging pulmonary artery at the upper left cardiac border; **b**: Right heart catheterization revealed a lager VSD; **c** and **d**: Pulmonary arteriography showed that the degree of pulmonary capillary filling decreased. PAH: pulmonary arterial hypertension; VSD: ventricular septal defect; PA: pulmonary artery; RPA: right pulmonary artery; LPA: left pulmonary artery; RILA: right inferior lobe artery

An 8-year-old girl presenting with cyanosis and exertional dyspnea was referred to our institution for minimally invasive transthoracic VSD closure. Physical examination revealed cyanosis with oxygen saturation in the upper and lower extremity of 86 and 84%, respectively. A loud P2 without a systolic murmur was audible at the third left intercostal space. Electrocardiogram demonstrated right ventricular hypertrophy. Chest X-ray evaluation will show cardiomegaly and a bulging pulmonary artery at the upper left cardiac border (Fig. 2a, arrow). An echocardiogram revealed a non-restrictive VSD (20 mm) and PAH (60 mmHg). A right heart catheterization revealed a lager VSD (Fig. 2b) and severe fixed PAH with a mean PAP of 86 mmHg and PVR of 18 Woods units. Pulmonary arteriography showed that the degree of pulmonary capillary filling decreased (Fig. 2c and d). Since the patient had fixed PAH, the surgical correction was not performed, and then was referred to medical treatment.

### PDA associated PAH

**Fig. 3:**
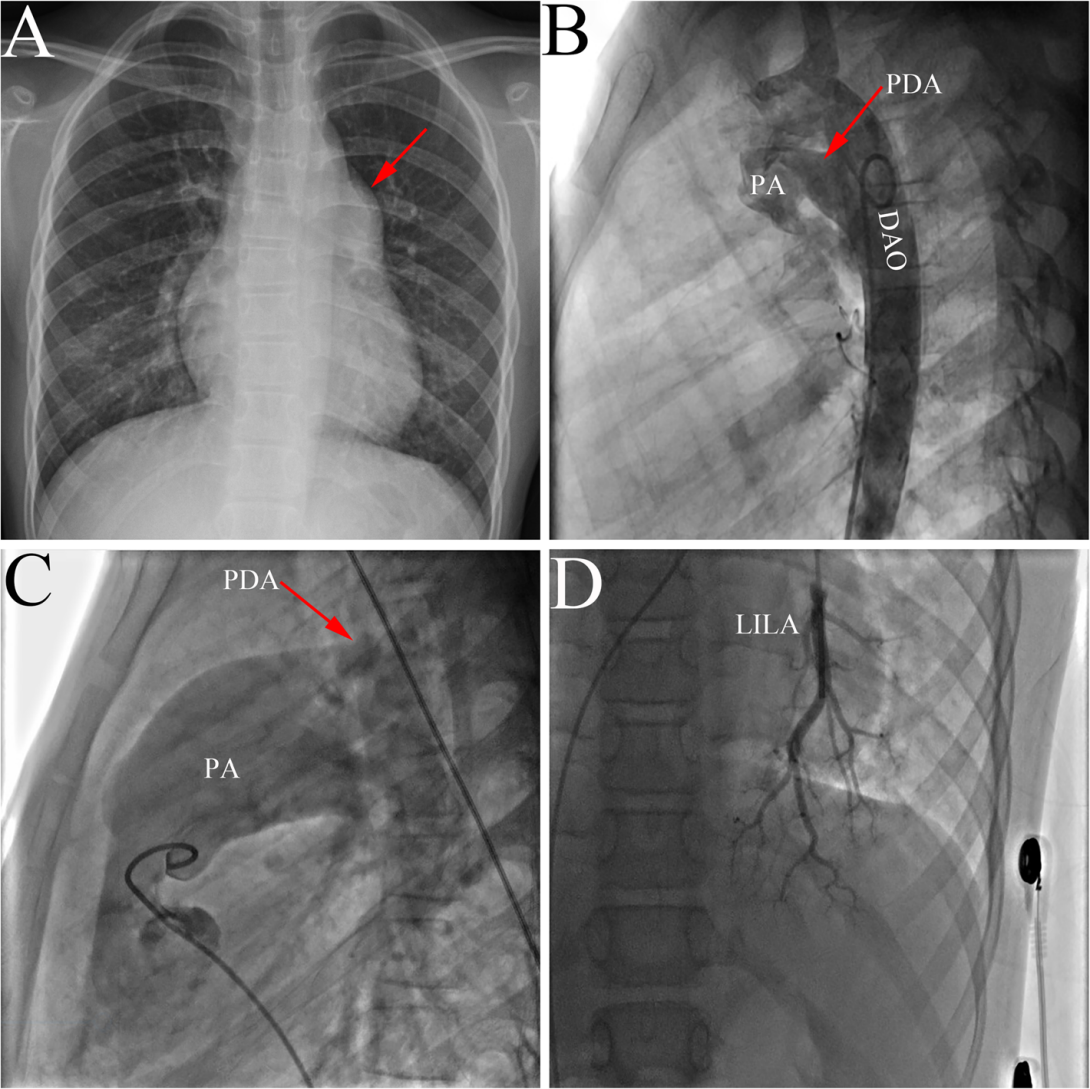
The representative image of PAH-PDA. **a**: Chest X-ray showed a bulging pulmonary artery at the upper left cardiac border; **b**: Descending aortography revealed a large PDA; **c** and **d**: Pulmonary arteriography showed a large PDA and decreased pulmonary capillary filling.. PAH: pulmonary arterial hypertension; PDA: patent ductus arteriosus; PA: pulmonary artery; DAO: descending aorta; LILA: left inferior lobe artery

A 12-year-old Chinese girl with a 10-year history of heart murmur and differential cyanosis was referred to our institution for minimally invasive transthoracic PDA closure. Physical examination revealed differential cyanosis with oxygen saturation in the upper and lower extremity of 95 and 86%, respectively. A loud P2 was audible at the second left intercostal space. Electrocardiogram demonstrated right ventricular hypertrophy. Chest X-ray evaluation will show cardiomegaly and a bulging pulmonary artery at the upper left cardiac border (Fig. 3a, arrow). An echocardiogram revealed a large PDA (15 mm) and PAH (60 mmHg). Descending aortography revealed a large PDA between the pulmonary artery and the descending aorta (Fig. 3b, arrow). A right heart catheterization revealed severe fixed PAH with a mean PAP of 71 mmHg and PVR of 12 Woods units. Pulmonary arteriography showed that a large PDA (Fig. 3c, arrow) and the degree of pulmonary capillary filling decreased (Fig. 3d). Since the patient had fixed PAH, the surgical correction was not performed.

## Discussion and conclusions

Pulmonary hypertension is by no means uncommon; on the contrary, it probably affects around 1% of the global population [8]. PAH is not in itself a diagnosis, but solely a hemodynamic state characterized by resting mean PAP of ≥25 mmHg. The term PAH describes a subgroup that is hemodynamically distinguished by precapillary pulmonary hypertension with elevated PVR [2], i.e., ≥25 mmHg with normal pulmonary arterial wedge pressure (PAWP) ≤15 mmHg and PVR > 240 dyn × s × cm^− 5^.

Blood flow and pressure are essential triggers for pulmonary vascular remodeling in CHD [9]. In CHD patients, the arteriopathy is triggered by increased pulmonary blood flow resulting from a left-to-right shunt due to an intracardiac or extracardiac defect. Early identification of the cardiac defect allows detection of no or mild (reversible) arteriopathy, and timely shunt closure can permanently repair the CHD. Long-standing overload of the pulmonary vasculature can result in the development of pulmonary vascular disease and an increase in pulmonary pressures. Non-restrictive, post-tricuspid shunts such as a VSD or PDA (high flow/high pressure) induce advanced PAH-remodeling frequently and rapidly, usually within a few years. In contrast, pre-tricuspid high flow/normal pressure lesions like ASD induce advanced remodeling only in 5–10% of the patients and generally only after two to four decades [10]. Increased flow, especially in combination with increased pressure, disturb blood flow throughout the pulmonary artery tree [11].

The cardinal symptom of PAH-CHD is progressive dyspnea, often accompanied by a prominent second heart sound, fatigue and exhaustion. The disease and associated symptoms are typically progressive, even though there is often a delay of many years or even decades between the onset of symptoms and therapy. However, the beneficial effects of shunt closure seem lost after a certain point of no return, after which even accelerated PAH progression may occur months to years after surgery. The assessment of reversibility is nowadays primarily based on clinical judgement and measurements of hemodynamic variables, which have limitations as surrogates for the stage of the arteriopathy. Techniques able to directly assess the pulmonary vasculature are still absent from clinical practice today.

Although echocardiographic diagnosis has been described, cardiac catheterization is still considered the gold standard method of diagnosis. Echocardiography is the mainstay of non-invasive diagnostic tools during the early screening that depicts intracardiac and extracardiac malformations, hemodynamic changes, right heart overload, PAH [12]. In recent years, cardiac CT has emerged as the standard of reference for identification and characterization of PAH-CHD. Cardiac CT allows a non-invasive display of pulmonary artery development, depicting a three-dimensional assessment of the anatomic relations between the pulmonary artery and adjacent structures [13, 14]. However, the classic measurement of the pressure of the pulmonary artery is by cardiac catheter of the right heart, and the gold standard of PAH is also this method. The main purposes are: (1) To accurately measure the right atrial and right ventricular pressure, PAP, PAWP, cardiac output and other hemodynamic parameters, which are helpful to assess the severity of PAH. (2) It is helpful to provide prognostic information and guide the correct treatment. (3) Cardiac catheterization is also helpful in excluding post capillary pulmonary hypertension while pulmonary angiography can be used to delineate the anatomy of the pulmonary arteries.

Early identification of the cardiac defect allows detection of no or mild (reversible) arteriopathy, and timely shunt closure can permanently repair the CHD. In our study, the surgical correction was not performed in all patients who had fixed pulmonary hypertension. According to current guidelines, assessment of reversibility is limited to hemodynamic variables: those in favor of reversible PAH-CHD are a left-to-right shunt and a PVR index < 4 Woods units. Shunt closure is contraindicated when the net shunt is directed right-to-left, and is discouraged when the PVR index is > 8 Woods units [1]. When the PVR index is between 4 and 8 Woods units, ‘individual patient evaluation in tertiary centers’ is advised [12]. Our initial experience confirms this. These recommendations however, are predominantly based on expert opinion and are hardly supported by data. In fact, in PAH-CHD, no prospective studies have yet identified reliable hemodynamic cut-offs that predict the reversal of pulmonary vascular disease and normalization of hemodynamics after cardiac correction.

In China, very few cases of irreversible PAH in simple CHD were reported in recent decades [14]. Considering that China has the biggest population in the world, the number of reported Chinese cases with PAH-CHD was far less than it should be. We believe that three main factors contribute to this. First, the lack of a stable and advanced medical system especially in Chinese rural areas causes misdiagnosis and limits surgical repair opportunities for simple CHD. Second, financial constraints prevent the referral of patients with CHD to a better hospital. Although the development of the Chinese economy has greatly alleviated the situation, there are still many patients with CHD undiagnosed and untreated in early life. At last, the lack of understanding and publicity about CHD, especially in remote areas of China, has led to neglect and misunderstanding of the disease and missed the best treatment period.

In conclusion, PAH in CHD can be reversed by early shunt closure, but this potential is lost beyond a certain point of no return. This article highlights the essence of enhancing the level of healthcare and services in Chinese rural areas. Failure to accurately and timely assess PAH will delay effective treatment past optimal treatment time, and even lead to death.

## Data Availability

The datasets used and/or analyzed during the current study are available from the corresponding author on reasonable request.

## Abbreviations

CHD: Congenital heart disease
PAH: Pulmonary arterial hypertension
TTE: Transthoracic echocardiography
CTA: Computed tomographic angiography
VSD: Ventricular septal defect
ASD: Atrial septal defect
PDA: Patent ductus arteriosus
PAP: Pulmonary artery pressure
PVR: Pulmonary vascular resistance
